# Evaluation of lower urinary tract disease in the Yogyakarta cat population, Indonesia

**DOI:** 10.14202/vetworld.2020.1182-1186

**Published:** 2020-06-25

**Authors:** Alfarisa Nururrozi, Yanuartono Yanuartono, Prisyarlinie Sivananthan, Soedarmanto Indarjulianto

**Affiliations:** Department of Internal Medicine, Faculty of Veterinary Medicine, Universitas Gadjah Mada, Yogyakarta, Indonesia

**Keywords:** cats, feline lower urinary tract disease, Yogyakarta

## Abstract

**Background and Aim:**

This paper reports a retrospective study performed in 185 cats diagnosed with feline lower urinary tract disease (FLUTD). The analyzed population involved feline patients at the Veterinary Clinic of Universitas Gadjah Mada, Indonesia. This research aimed to evaluate the clinical indications and causes of FLUTD in the Yogyakarta cat population.

**Materials and Methods:**

The medical data of all feline patients were obtained to conduct this study. FLUTD diagnoses were based on physical examinations, urinalyses, ultrasound examinations, and bacterial cultures. Only cats with a complete examination were used in the study. The clinical signs were evaluated and accompanied by the results of laboratory tests in cats that showed symptoms of FLUTD. The medical history of all feline patients was analyzed thoroughly. Most of the feline’s urine samples were collected by catheterization.

**Results:**

The most commonly diagnosed of FLUTD in the Yogyakarta cat population were: feline idiopathic cystitis (FIC) (56%), urinary tract infection (UTI; 25%), urolithiasis (13%), urethral plugs (UP) (4.9%), and neoplasia (0.4%), respectively. The prevalence of UTI is higher than that reported in Europe or the US. Older cats more often show symptoms of UTI and neoplasia, whereas young cats more often show symptoms of FIC and UP. The prevalence of male cats experiencing FLUTD in Yogyakarta is much higher than female cats.

**Conclusion:**

The incidence rate of FLUTD cases in Yogyakarta is related to age and sex. The results of this study are similar to those of the previous research studies conducted in other countries.

## Introduction

Feline lower urinary tract disease (FLUTD) is considered on problems related to a cat’s urethra and urinary bladder [1,2]. FLUTD is a broad terminology that involves many disorders, including feline ­idiopathic cystitis (FIC), urethral obstructions, urolithiasis, urinary tract neoplasia, and urinary tract infection (UTI) [3]. The data obtained for this study correspond to complaints (signalment) from the owner who commonly observed signs of blood in the urine (hematuria), urinary straining (stranguria), inappropriate urination (periuria), and dysuria [4]. According to the previous research, cats with any problems in the urinary tract show identical symptoms [1,3].

The epidemiology data in FLUTD cases have not been reported in Yogyakarta. Researchers believe that the most frequent type of FLUTD is FIC [1,5,6]. FIC is demonstrated between 55% and 63% of the cats with FLUTD [7]. The other causes of FLUTD are urinary calculi, urethral plugs (UP), and UTIs. Neoplasias are categorized as being one of the less common causes of FLUTD [3]. Based on the literature, there are similar results in the prevalence of FIC, UP, urolithiasis, and neoplasias [6]. Conversely, there are differences in UTI cases in European countries, which are reported to vary between 8% and 20%. These are higher than reports in the US [5]. The epidemiology data on FLUTD cases have not been reported in Yogyakarta.

The previous study had the highest occurrence of UP observed in cats ranging in age from 2 to 7 years old [5,6]. Cats that ranged in age from 4 to 10 years old had a higher risk of urolithiasis and idiopathic cystitis, whereas cats 10 years and older were significantly exposed to risks of UTI and neoplasia [5,6]. The previous research reported that FIC was more convenient to diagnoses in young cats, whereas the risk of UTI, urolithiasis, and neoplasia increased with older age [1,3,8]. Certain common breeds were reported to experience lower urinary tract disease more frequently, such as Persian, Himalayan, and Russian Blue. Some of these breeds are considered to have a predisposing factor to the formation of uroliths so that urinary tract obstruction occurs. Abyssinian cats were commonly predisposed to bacterial UTI [8].

Castrated males had an increased risk of UTI, urolithiasis, and neoplasia compared with spayed females [5,8,9]. Sexually intact females had a decreased risk for each cause of FLUTD except neurogenic disorders and iatrogenic injuries. Castration and spaying are considered risk factors associated with the inhibition of urethral growth, induction of weight gain, and a sedentary lifestyle [5,6]. Although the disease occurs in both the sexes, FLUTD is rare in females common in male cats, due to the anatomy of the penile urethra [2,9].

Urinary tract diseases could be accurately diagnosed with the required access to the medical record and clinical and laboratory examinations [2]. The objective of this retrospective study was to evaluate the clinical symptoms, factors (age, sex, breed, and weight), and the type of FLUTD found in Yogyakarta based on the data supplied by the veterinary clinic.

## Materials and Methods

### Ethical approval

The research for this study was conducted at the Veterinary Clinic, the Department of Internal Medicine, Faculty of Veterinary Medicine, Universitas Gadjah Mada. All data and samples that were collected from cats that were used for research have been permitted by the owner through signing an informed consent form.

### Sample collection

A total number of 185 cats were selected from the Yogyakarta Province. This study was undertaken from January 2017 to July 2019. Cats with clinical signs of hematuria, stranguria, pollakiuria, periuria, and dysuria were used in this research. The cats used must not have received prior medical treatment. The data obtained were the animal’s age, breed, sex, type of clinical signs, and frequency of urination. Owners were asked to provide additional information regarding the duration of the symptoms, the number of cats in the household, and other factors thought to cause stress. FLUTD diagnoses are based on the physical examination, urinalysis, ultrasound examination, and bacterial culture. Only cats with a complete clinical and laboratory examination were used in the study. The data on age, sex, breed, and bodyweight of cats that were diagnosed with FLUTD were collected.

### Procedures

Urinalyses were required to determine urinary abnormalities, urine biochemistry, sediment evaluation, and calculi type for examination. Ultrasonography was performed on patients to detect the presence or absence of calculi in the urinary tract and the signs of inflammation [10]. A blood examination was conducted to determine the status of urea in the blood and to assess the kidney function of patients. The complete urinalysis using reagent strips included the determination of glucose, bilirubin, ketone bodies, and hemoglobin content of urine, and conducted a protein test. Urine pH was determined using a pH meter, whereas urine specific gravity (USG) was measured with a hand refractometer [11].

The urine from suspect samples was tested by cytological and microbiological culturing. Urine samples were centrifuged, and the sediment was examined for the presence of red and white blood cells. Crystal formations were observed and evaluated. Microbiological tests were done on suspect samples obtained by catheterization and grown on mannitol salt agar, MacConkey agar, and blood agar (containing 5% sheep blood). The cultures were incubated at 37^o^C for 24-48 h [12-14].

The studied patients were categorized into FIC, urolithiasis, UP, UTI, and neoplasia according to their respective diagnoses. UTI is diagnosed by significant bacterial growth from urine samples [13-15]. UP are diagnosed with the detection of plugs that cause ­urethral obstruction. A urolith is diagnosed using ultrasonography or X-ray. Neoplasia is diagnosed ultrasonographically by the identification of a mass lesion. FIC is diagnosed by eliminating the other specific possibilities. FLUTD diagnostic methods in this study are based on the parameters used by Dorsch *et al*. [5] (Table-1).

**Table-1:** Definitions and diagnostic methods for various types of FLUTD [5].

Type FLUTD	Methods
FIC	Eliminate the other specific causes possibilities
UTI	High number bacteria (>10^3^ CFU / ml) in urine culture in growth media
UP	The obstruction of the urethra caused by the plug identified on catheterization with/without crystalluria on urine sediment
Urolithiasis	Bladder/urethral stone detected on radiography and/or ultrasonography
Urinary tract neoplasia	The lesion was identified by ultrasound examination

FIC=Feline idiopathic cystitis, UTI=Urinary tract infections, UP=Urethral plugs

### Statistical analysis

Analyses were conducted using SPSS statistic programs version 16 (IBM Corp., NY, USA). Descriptive statistics (mean, standard deviation, median, and range) were calculated. Comparison of continuous parameter was performed using one-way ANOVA and Tukey’s comparison test. Statistical significance was set at p<0.05. Statistical analyses were only performed with minimum of ten cats in on each group.

## Results

The incidence of FLUTD was obtained from 185 patients during 2017-2019 from the Veterinary Clinic of Universitas Gadjah Mada, Indonesia. Precise medical records, including clinical examinations, ultrasound tests, and blood and urine tests, were obtained from the clinic. The indications regarding the animal’s behavior during urination was provided by the owner. The clinical signs showed by the majority of patients included 84 cats (45.3%) with stranguria, 22 cats (11.9%) with pollakiuria, 11 cats (6.0%) with dysuria, and six cats (3.2%) with periuria. Macroscopic hematuria was observed in 40.4% of patients (Table-2).

**Table-2:** Clinical signs analyses of cats with FLUTD (n [%] of cats).

	All cats	FIC	UTI	UP	Urolithiasis	Neoplasia
Total	185 (100)	103 (56.1)	47 (25.3)	9 (4.9)	24 (13.0)	1 (0.4)
Hematuria	75 (40.4)	43 (41.3)	16 (33.3)	1 (14.3)	14 (56.8)	1 (100)
Stranguria	84 (45.3)	19 (18.8)	40 (86.1)	6 (64.3)	23 (97.3)	1 (100)
Pollakiuria	22 (11.9)	12 (11.3)	1 (1.4)	0 (0.0)	10 (40.5)	0 (0.0)
Dysuria	11 (6.0)	3 (2.5)	1 (1.4)	8 (85.7)	0 (0.0)	0 (0.0)
Periuria	6 (3.2)	3 (3.1)	1 (2.8)	0 (0.0)	1 (2.7)	1 (50.0)

FIC=Feline idiopathic cystitis, UTI=Urinary tract infections, UP=Urethral plugs

Hematuria was observed most in FIC, with 57% compared with other clinical signs. In contrast, stranguria was noted to be the highest clinical sign in UTI, UP, and urolithiasis and was diagnosed in 40 (86.1%), 6 (64.3%), and 23 (97.3%) of total cases, respectively.

The epidemiologic data, including sex, breed, and age of cats diagnosed with FLUTD, are presented in Tables-3 and 4. The population studied comprised 83 (44.9%) Persian, 65 (35.4%) domestic short hair, 14 (7.7%) mixed breed, 6 (3.5) Himalayan, 5 (2.5%) Angora, 3 (1.4%) British shorthair, 5 (2.8%) Maine coon, and 3 (1.8%) unknown breeds of cats. FIC and urolithiasis were diagnosed more in the Persian breed. Domestic cats had a prevalence of 65 (35.4%) patients in total, with 58.3% being the highest recorded for UTI. The average age of cats with FLUTD in Yogyakarta is 2.4 years; however, the age variation was high from 4 months to 12 years. The mean age in UP (0.9 years) was the youngest, whereas the mean in the case of neoplasia (9.8 years) was the oldest. The average age of UTI and neoplasia was significantly older than the other group (p<0.05) with an average age of 9.1 and 9.8 years, respectively. The averages in FIC and UP were younger than other FLUTD cases with an average age of 2.0 and 0.9 years, respectively. The data of age and breeds and sex in 185 cats with FLUTD are shown in Tables-3 and 4.

**Table-3:** Age and breed evaluation in 185 cats suffering with FLUTD.

	All cats (n=185)	FIC (n=103)	UTI (n=47)	UP (n=9)	Urolithiasis (n=24)	Neoplasia (n=1)	p-value
Age (year)	(0.2-12) 2.4	(0.6-7) 2	(5.3-11.5) 9.1	(0.8-1) 0.9	(2.9-8.6) 6.8	(6.5-12) 9.8	<0.05^1^
Persian	83 (44.9)	55 (53.8)	8 (16.7)	4 (42.9)	14 (59.5)	1 (100)	<0.05^2^
Domestic	65 (35.4)	28 (27.5)	3 (58.3)	2 (21.4)	8 (32.4)	0 (0.0)	
Mixed breed	14 (7.7)	6 (6.3)	7 (13.9)	1 (14.3)	1 (5.4)	0 (0.0)	
Himalayan	6 (3.5)	3 (3.1)	2 (4.2)	0 (0.0)	1 (5.4)	0 (0.0)	
Angora	5 (2.5)	3 (2.5)	1 (2.8)	1 (7.1)	0 (0.0)	0 (0.0)	
British shorthair	3 (1.4)	1 (1.3)	1 (1.4)	1 (7.1)	0 (0.0)	0 (0.0)	
Maine coon	5 (2.8)	3 (3.1)	1 (1.4)	1 (7.1)	1 (2.7)	0 (0.0)	
Unknown breed	3 (1.8)	3 (2.5)	1 (1.4)	0 (0.0)	0 (0.0)	0 (0.0)	

FIC=Feline idiopathic cystitis, UTI=Urinary tract infections, UP=Urethral plugs.

1 Cats with UTI or neoplasia were significantly older than FIC, UP, and urolithiasis (p<0.05).

2 Persian cats were significantly suffering FIC and urolithiasis than other FLUTD

**Table-4:** Sex analyses of cats with FLUTD (n [%] of cats).

	All cats (n=185)	FIC (n=103)	UTI (n=47)	UP (n=9)	Urolithiasis (n=24)	Neoplasia (n=1)	p-value
Males (%)	82.1	77.5	97.2	92.8	67.6	100	<0.05^1^
Females (%)	17.9	22.5	2.8	7.2	32.4	0.0	

FIC=Feline idiopathic cystitis, UTI=Urinary tract infections, UP=Urethral plugs.

1 The proportion of male cats suffering FLUTD was significantly higher than female (p<0.05)

The examined population consisted of 82.1% males and 17.9% females. Male cats were more common than female cats in all types of FLUTD, including FIC, UTI, UP, urolithiasis, and neoplasia. The highest percentage of female cats was found in urolithiasis (32.4%) and FIC (22.5%). The sex data of 185 cats with FLUTD are shown in Table-4.

## Discussion

This study describes FLUTD that was found in 185 cats of the Yogyakarta cat population. The data accumulated provide information that the highest rate of FLUTD in Indonesian cats was FIC. The prevalence observed in different countries by other researchers showed that FIC also has a high percentage. In a study conducted in the Norwegian cat population during 2003-2008, the prevalence of FIC was reported as 55.5% [6]. This result is similar to the finding of other research that reported the incidence of FIC as being 50%-63% of FLUTD [3-6,9,16].

The second significant cause of FLUTD reported in this study was UTI, with a rate of 25.3%. The percentage of UTI cases from this study was higher than that reported in the United States, which is <3% of that reported in Poland which is approximately 15%. The high numbers of UTI cases in this study are first-opinion cases. The high infection in this study may be because most cats in the study are stray cats that are more likely to be infected than indoor cats.

Lekcharoensuk *et al*. [8] reported major risk factors of UTIs, including old age, perineal urethrostomy, low USG, and previous catheterization history. The bacteria often found in feline UTI tests are *Escherichia coli*, *Enterococcus* spp., and *Staphylococcus felis* [15]. Different researchers found that bacterial UTI in cats are generally seen in female cats [16]. However, in this study, male cats showed higher UTI cases than females. UTIs can also occur in healthy male cats that are catheterized, with the risk of infection increases with the duration of catheterization [13,17].

The mean age of cats suffering with UTI in this recent research (9.1) is slightly higher compared with other studies (6.7, 5.1, and 5.6 years, respectively) [5,6,8,18]. The average age of cats with FIC, UP, and urolithiasis was 2.0, 0.9, and 6.8 years. The mean age of cats suffering from neoplasia is significantly older than the other group (9.8 years). The previous studies have shown that cats experience FLUTD at an older age (>10 years), the many of which (around 45%) suffer from UTI, whereas 17% suffer from UTI, which progresses to urolithiasis. These data suggest that a routine urine culture and sensitivity test should be performed in older cats with signs of FLUTD.

Based on the results of complete urinalysis combined with ultrasound or radiographic examination, 13% of 185 cats suffering from FLUTD showed urolithiasis. In urolithiasis, most (97.3%) cats showed symptoms of urination difficulties. The formation of crystal struvite and oxalates was observed in most ­urolithiasis cases. In this study, 40% of urine samples contained high numbers of red blood cells under microscopic analyses. Based on other research conducted by Lew-Kojrys *et al*. [9], 97% had hematuria in the urine samples. Later, in the year 2011, an 83% prevalence of hematuria was found by Saevik *et al*. [6].

The previous studies reported that oxalate crystals and stones are more often found in Himalayan, Persian, Burmese, and Russian blue cats. Genetic factors may also contribute to an increased risk of calcium oxalate urolith formation. In this study, a total of 83 patients were Persian cats, with 60% having identified urolithiasis [8]. The second highest prevalence was seen in domestic breeds, with 58% having feline UTI. Most cats in our sample were indoor and outdoor cats that had higher risks of infection before drinking water from flower bowls and drains with bacteria infestations [13].

Similar to other studies, the incidence of FLUTD is more often found in male cats [19]. In this study, 82.1% of FLUTD cases were found in male cats, whereas 17.9% were found in female cats. In contrast to the previous studies by Dorsch *et al*. [5] and Saevik *et a*l. [6], who categorized cats as castrated or spayed, no clustering was done in this study. This is because many cats are stray cats that are not known with certainty, whether they have been castrated.

## Conclusion

This research reports that the most significant FLUTD diagnosed in this research was FIC. This result is similar to that reached by the report of European and US studies. The prevalence of UTI is higher than that reported in other countries. The incidence rate of FLUTD cases in Yogyakarta is related to age and sex. Older cats more often show symptoms of UTI and neoplasia, whereas young cats more often show symptoms of FIC and UP. The prevalence of male cats experiencing FLUTD in Yogyakarta is much higher than female cats.

## Authors’ Contributions

The research was determined, managed, and supervised by SI. AN and PS took samples, recorded samples, and performed sample analysis. AN and YY identified and analyzed data. All authors have observed, wrote, and ratified the final manuscript.
